# Non-Linear Heart Rate Variability and Risk Stratification in Cardiovascular Disease

**Published:** 2005-07-01

**Authors:** Phyllis K Stein, Anand Reddy

**Affiliations:** Washington University School of Medicine, St. Louis, MO, USA

**Keywords:** heart rate variability, risk stratification, non linear, post myocardial infarction, congestive heart failure

## Abstract

Traditional time and frequency domain heart rate variability (HRV) have cardiac patients at risk of mortality post-myocardial infarction. More recently, non linear HRV has been applied to risk stratification of cardiac patients. In this review we describe studies of non linear HRV and outcome in cardiac patients. We have included studies that used the three most common non-linear indices: power law slope, the short term fractal scaling exponent and measures based on Poincare plots. We suggest that a combination of traditional and non-linear HRV may be optimal for risk stratification. Considerations in using non linear HRV in a clinical setting are described.

The association between traditional time and frequency domain measures of heart rate variability (HRV) and cardiovascular outcomes has been the subject of numerous investigations. Although there were earlier reports suggesting the potential clinical importance of HRV, the well-known paper published by Kleiger and colleagues reporting the results of the Multi-Center Post-Infarction Project (MPIP) in 1987 can be viewed as the beginning of serious interest in HRV among cardiologists [1]. This paper reported the remarkable independent association of a single, time domain HRV measure, SDNN, the standard deviation of all normal-to-normal (NN) interbeat intervals over 24-hours and risk of mortality 1-year post-MI. With the publication of this paper, the race was on and investigators tried to determine whether other time domain HRV variables were, perhaps better predictors of outcome. By 1991, frequency domain measures, based on the fast Fourier transform or similar mathematical analyses, had been applied to 24-hour Holter recordings, with seemingly even better results [2]. Large datasets of Holter recordings began to be collected, especially by the group at St. George’s in London who developed their own geometric measures of HRV that could be applied to Holter recordings without the requirement of painstaking and exacting characterization of all interbeat intervals [3]. By the mid-90’s, there were a number of studies verifying the predictive value of HRV in various cardiac patient populations, and the Task Force of the European Society of Cardiology and the North American Society of Pacing and Electrophysiology had issued a set of formal recommendations for the measurement and clinical uses of HRV [4].

At about that time, investigators began to consider whether measures of HRV derived from non-linear mathematics might have some application to clinical populations. It is beyond the scope of this review to detail the various methods that were applied. Rather, we will concentrate in detail on the three best-known: the power law slope, measures derived from the Poincaré plot and the short-term fractal-scaling exponent. In the sections below we will discuss some of the significant investigations in the field. Table 1 provides a more complete listing of studies in the field.

## Power Law Slope

**Figure 1:**
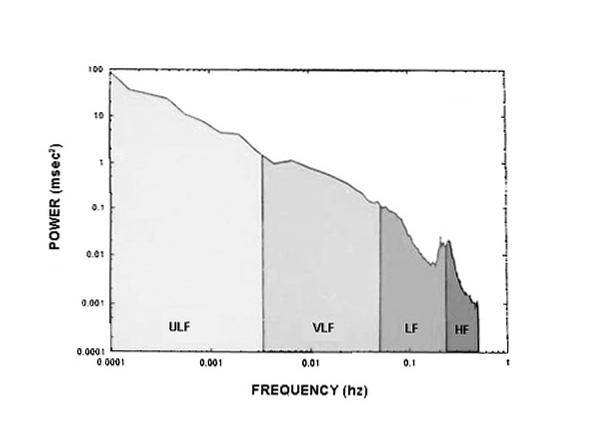
Log: log plot of the HRV power spectrum over 24 hours. The region between 0.01 and 0.0001 Hz is used to calculate power law slope

When HRV is analyzed in the frequency domain, the variance of the signal is decomposed into its sinusoidal components at various underlying frequencies. This can be visualized in a plot of the HRV power spectrum (see Figure 1) in which the area under the curve at each frequency (on the x-axis in Hz) is equal to the variance explained at that frequency. In 1982 Kobayashi and Musha reported on the frequency dependence of the HRV power spectrum a normal young man [5]. That is, they noted that when the power spectrum is plotted on a log:log scale, as in Figure 1, at least below 10-2 Hz, the amount of power increased, linearly, as the underlying frequency decreased (called a 1/f relationship). The slope of this regression line is now referred to as the power law slope, and its intercept with the y-axis is the power law intercept. In non-linear terminology, this meant that fractal scaling properties of longer-term HRV were self-similar on a scale of minutes to hours.

**Figure 2:**
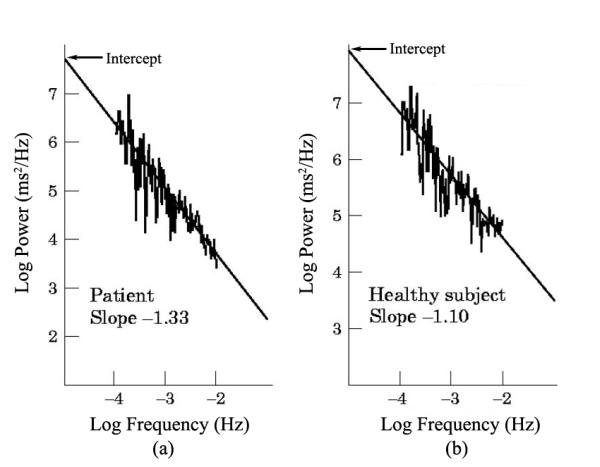
Examples of the power law slope in a) a patient with cardiac disease. And b) a healthy person

The potential prognostic value of the power law slope was reported in 1996 when Bigger and his co-workers re-analyzed data from MPIP [6]. In addition, they compared power law slope and intercept between N=274 MPIP patients (patients with a recent myocardial infarction), 274 healthy persons and 19 people with heart transplants. They concluded that MI caused a relative denervation of the heart (steeper slope, decreased intercept) that was significantly more adverse among heart transplant patients. This is illustrated in Figure 2 which compares power law slope for a healthy subjects and one with severe cardiac disease. Individually and especially combined, power law regression parameters were stronger predictors of death from any cause or arrhythmic death than traditional measures of time or frequency domain HRV.

In a subsequent prospective study by Huikuri and his co-workers, N=347 people >65 years of age were followed for 10 years [7]. Power law slope (<-1.50) was the best univariate predictor of all cause mortality (odds ratio, 7.9; 95% CI 3.7 to 17.0; p<0.0001). Decreased power law slope predicted both cardiac and cerebrovascular death, suggesting that altered long term behaviour of HR reflects an increased risk of vascular causes of death, rather than being a marker of any disease or frailty leading to death.

Similarly, HRV indices, including power law slope was compared in 2 groups of patients after acute myocardial infarction, one with normal and one with reduced ejection fraction. A more negative power law slope was observed in patients with reduced ejection fraction after myocardial infarction but not in those with a normal ejection fraction [8]. This study was small (N=35) and no relationship with outcome was reported.

## DFA1 (Short-Term Fractal Scaling Exponent)

In 1995, CK Peng and colleagues proposed a novel non-linear measure of HRV called the short-term fractal scaling exponent [9]. Unlike many of the other proposed non-linear measures, this new measure was relatively straightforward to compute and not hampered by any mathematical assumptions. The mathematical algorithm for calculating the short-term fractal scaling exponent (referred to as DFA1, alpha-1 or α1) can be downloaded from PhysioNet www.PhysioNet.org. DFA1 has been calculated from all interbeat intervals, regardless of their aetiology, and it has been calculated from normal-to-normal interbeat intervals only. DFA1, which can be calculated from a minimum of 1000 beats and then averaged, reflects the amount of randomness in the heart rate time series, with the lowest values (~0.5) associated with a completely random time series and the highest values (1.5) associated with a time series that is totally correlated. It is becoming clear that increased randomness of heart rate patterns, i.e., a high degree of sinus arrhythmia of non-respiratory origin, is associated with a worse prognosis [10].

It should be noted that the algorithm for the calculation of DFA1 has recently been improved, and although values calculated using the old and new algorithms correlate >0.95, the old value for normal DFA1 was a bit over 1 and the new one is about 1.2. All of the studies referred to below use the old algorithm but future studies will increasingly use the newer one.

In the TRACE (**TR**Andopril **C**ardiac **E**valuation study) which enrolled 159 patients with acute MI and with LV wall motion score <1.2 or LVEF <35%, Holter recordings were obtained 3.7 days post-MI. Patients were followed for up to 4 years, at which time 45% had died. Among all analysed variables, reduced short-term fractal scaling exponent (α1<0.85) was the best univariable predictor of mortality (relative risk 3.17, 95% CI 1.96-5.15, p<0.0001) with positive and negative predictive accuracies of 65% and 86% respectively. Even after adjustment for clinical covariates and LV function, in the Cox proportional hazards analysis, decreased α1 was an independent predictor of mortality (p<0.001) [11].

More recently, α1 was shown to predict mortality in patients with heart failure. In a sub study of the DIAMOND CHF trial which enrolled patients with left ventricular wall motion index ≤1.2 (equivalent to ejection fraction 35%), N=499 eligible patients were followed up for mean period of 665 ± 374 days, during which time N=210 died. After adjustment for age, functional class, medication and left ventricular ejection fraction in the multivariate proportional hazards analysis, decreased α1 predicted mortality (RR=1.4, 95%CI 1.0-1.9, p<0.05). HRV was a better predictor of outcome among those with moderate heart failure (Class II) but HRV was not an independent predictor of mortality among those with severe heart failure (class III or IV).

Recently Laitio and his co-workers [12] reported that reduced alpha1 is predictive of postoperative myocardial infarction in elderly patients having emergent surgery for traumatic hip fracture. N=32 patients, aged 60, with preoperative nighttime and daytime Holter recordings were studied. The short term fractal scaling exponent of RR intervals was assessed from 2:00 AM to 5 AM and 7AM to 12 noon for each patient. Preoperative α1 was significantly lower during the nighttime compared with the daytime (0.92±0.08 vs. 1.03±0.06, p=0.002) in patients with postoperative MI. Results suggest that preoperatively decreased night time fractal correlation properties of HR dynamics may identify elderly patients at risk for developing myocardial ischemia in the early postoperative phase after nonvascular surgery.

## Poincaré Dimension

**Figure 3:**
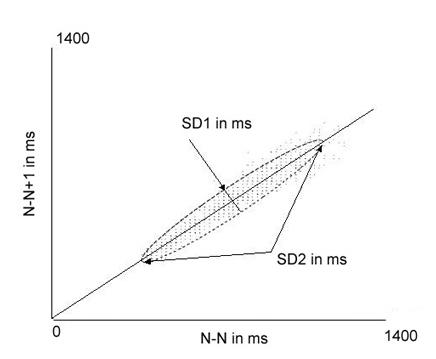
Example of a Poincaré plot showing the calculation of SD1 and SD2 from an ellipse fitted to the plot

The Poincaré plot is a graphic method for representing the underlying structure of the R-R interval time series. In a Poincaré plot, also known as a return map plot, the time between each pair of beats is plotted against the time between the next pair of beats, i.e., the x, y co-ordinates of each point are rr_i_, rr_i+1_. An example is see in Figure 3. As is the case with the short-term fractal scaling exponent, some investigators plot every interbeat interval in the heart rate time series and others use only the normal-to-normal intervals. When all R-R intervals are plotted, the assumption is made that only the beats in the central portion of the plot are normal and that outlier beats are ectopic. Plots of morphologically normal-to-normal interbeat intervals have proved that this assumption is simplistic and misleading [10].

In one of the earliest studies involving Poincaré plots in a cardiac patient population, Woo et al [13] constructed Poincaré plots of sinus R-R intervals from 24-hour Holter recordings in N= 24 healthy control subjects and N=24 patients with heart failure. In the control group, plots had a comet shape resulting from an increase in beat-to-beat dispersion at slower heart rates. No heart failure patient displayed a comet-shaped pattern. Instead, three distinctive patterns were identified: (1) a torpedo shaped pattern resulting from low R-R interval dispersion over the entire range of heart rates, 2) a fan-shaped pattern resulting from restriction of overall R-R interval ranges with enhanced dispersion and 3) complex pattern with clusters of points characteristic of sudden changes in R-R intervals. Thus, the Poincaré plots revealed a complexity in heart rate patterns, which could not be perceived from standard heart rate variability measures like SDNN (the standard deviation of all normal-to-normal interbeat intervals). Indeed SDNN was not different in patients with different HR patterns. However, serum norepinephrine levels were significantly higher among heart failure patients with complex Poincaré plots, suggesting that these plots are a marker for increased sympathetic activation and might provide additional prognostic information and insight into autonomic alterations in patients with heart failure [14].

## Quantification of Poincaré Plots

**Figure 4:**
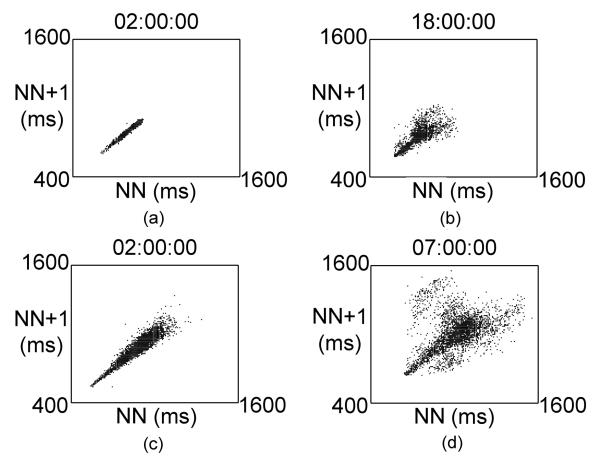
Representative one hour Poincaré plots. Figures 4a) and 4c) show normal HRV patterns. Figures 4b) and 4d) show abnormal, complex HRV patterns

We previously alluded to the presence of increased randomness in heart rate patterns and their potential as a marker of increased risk. As can be appreciated from Figure 3, because each point on the plot represent the relationship of one beat to the next, increased randomness of heart rate patterns can easily be recognized in the Poincaré plot as well.  Figure 4 shows examples of normal and abnormal heart rate patterns seen on hourly Poincaré plots. These plots illustrate that an increased dispersion of points results in an increase in the relative width of the plot compared with its length. The non-linear index SD12 quantifies the shape of the Poincaré plot. SD12 is the ratio of the lengths of the axes of an imaginary ellipse which has its center at the average RR (or NN) interval of the time series and is fitted to the Poincaré plot. As shown in Figure 3, the longitudinal axis of this ellipse is along a line beginning at the origin and with a slope of 1 and has a length called SD2. SD2 reflects intermediate-term variability. The length of the transverse axis of the ellipse is SD1 and reflects short-term variability. SD12 is the ratio of these measures.

In our lab, HRV from 740 tapes recorded before antiarrhythmic therapy in the Cardiac Arrhythmia Suppression Trial was studied [15]. CAST was an historic post MI study with impaired left ventricular ejection fraction and high grade ventricular arrhythmias that were suppressed with one of three antiarrhythmic therapies (encainide, flecainide or moricizine) [16]. Holter recordings were obtained at a large range of times post MI. Subjects in the HRV substudy were patients with ventricular premature contractions (VPCs) suppressed on the first randomised antiarrhythmic treatment. They were 70±121 post MI and follow up was 362±241days (70 deaths). Traditional time and frequency domain HRV were determined from the qualifying, pre-randomization Holter recording. Non-linear HRV measures included short-term fractal scaling exponent and SD12, each calculated from an average of 1000 normal-to-normal interbeat intervals and power law slope calculated from the entire recording. As mentioned, this group of patients was at a broad range of times post-MI whereas in the studies discussed earlier HRV was measured shortly after MI. As a result, some patients had undergone CABG surgery between their MI and their first Holter recordings. Decreased HRV among post-CABG patients is caused by the surgery itself and has been shown to be of no prognostic value. Therefore, post-CABG patients were excluded from the analysis [17]. Similarly, in the CAST population, the decreased HRV associated with diabetes was not prognostic. Thus, when the analysis was limited to those without CABG surgery post-MI or diabetes, power law slope had no association with mortality. A significant association was seen for DFA1 (p<0.001). However, increased SD12 had the strongest association with mortality. Importantly, however, in this study, we showed that traditional time and frequency domain HRV and non-linear HRV were *independent* predictors of mortality. That is, when all clinical and demographic covariates were considered, the combination of increased SD12 (<55%), decreased ultra low frequency power (a measure of the circadian rhythm of HR), history of prior MI and history of CHF identified the patients at highest risk of mortality.

We also determined the predictive value of DFA1 in association with traditional frequency domain HRV in the Cardiovascular Health Study, a prospective, population-based study of risk factors for heart disease and stroke among community-dwelling adults aged >65 year [18]. Of these participants, 1227 had 24-hour recordings eligible for frequency domain and non-linear analysis that were obtained at the beginning of the study. After 8 years of follow up, 267 had died. On univariate analysis, decreased values for all frequency domain HRV indices except for high frequency power (HF, a measure of the power of vagally-modulated respiratory sinus arrhythmia), and also decreased DFA1, were significantly associated with mortality. When HRV indices were combined, even after adjustment for age, gender, presence of subclinical or clinical cardiovascular disease and diabetes, the combination of decreased DFA1 and decreased HF was the strongest predictors of mortality. In the adjusted model, the risk ratio for DFA1 was 0.15 (95% CI 0.08-0.29) and for (ln) HF it was 0.78 (95% CI 0.069-0.88) all p<0.001. We hypothesize that HF power is significantly exaggerated by sinus arrhythmia of non-respiratory origins, thus eliminating its association with mortality. However, when DFA1 is added to the model, it essentially controls for this randomness, permitting the association of decreased vagal modulation of HR and mortality to be determined.

## Practical Considerations

Both increased SD12 and decreased DFA1 reflect increased randomness in the heart rate time series and are therefore highly correlated. As mentioned, DFA1 is dependent on accurate Holter scanning, but SD12 is less sensitive to scanning error. This might help explain its better prognostic value in the CAST study, mentioned above; where Holters were scanned on older equipment where accurate labelling of each beat would have taken days in some cases. Also, Poincaré plots can be examined visually and additional insights gained from their structure. While most studies that have used Poincaré plots examined patterns from 24-hour recordings, 24-hours of data represents >100,000 beats and details patterns may be obscured by looking only at the entire recording. In our lab, we examine hourly Poincaré plots and have noted that periods of increased randomness of HR patterns can occur in recordings that have many hours of normal patterns. Finally, power law slope, because it is based on a regression line, is relatively insensitive to scanning errors, but its prognostic value has not been as consistently verified.

## Caveats and Conclusions

It is clear that non-linear HRV analysis has the potential to substantially improve the utility of HRV for risk stratification. The circumstances under which non-linear HRV is applicable have not been fully elucidated. Non-linear HRV is not available from commercial Holter scanning systems, but most export an annotated beat-to-beat file that, with a little bit of computer expertise, can be used to generate non-linear HRV indices. Considerable care must be taken to accurately label the morphology of each beat. The Holter scanning system must provide a way to ensure that the interbeat intervals reflect a consistent detection of beat onset at the same point of each QRS, or a least of each series of QRS. Unfortunately, at the moment this is often not the case. Filters that automatically label beats by prematurity are not sufficient to insure accurate enough scanning, certainly for calculation of DFA1, at least when it is based on normal-to-normal interbeat intervals. Computer technology is advancing rapidly. The need to risk-stratify patients for implantable cardiac defibrillators has enlivened the search for Holter-based risk markers of increased risk. Because of these factors, it is likely that future Holter scanners will have far more sensitive beat-detection and beat classification algorithms, making it possible to obtain sufficiently accurate scanning to permit routine calculation of non-linear HRV for risk stratification in the clinical setting.

## Figures and Tables

**Table 1:** Studies of Non-Linear HRV in Cardiac Patients

Reference	Population	Results/Conclusions
Brouwer et al., 1996 [19]	95 pts with HF, HRV and Poincaré plots from 24 hr holter recordings (Ibopamine Multicenter Trial study group)	Shape of Poincaré plots independent prognostic value in pts with mild to moderate HF
Bigger et al., 1996 [2]	(1) 715 pts with recent MI (2) 274 healthy pts (3) 19 pts with heart transplant (Multicenter Post Infarction Program)	MI or denervation of the heart causes a steeper slope and ↓ height of power law slope
Huikuri et al., 1999 [10]	446 with MI with ↓ LV function (EF<35%) F/U 685±360 days	Alpha-1 is the most powerful predictor of all cause mortality
Huikuri et al., 1998 [7]	Random sample of 347 patients of >65 yrs F/U for 10 yrs	Power law slope is a more powerful predictor of death than the traditional risk markers in elderly subjects
Kamen et al, 1995 [21]	Poincaré plot pattern to display beat to beat HRV data from 23 pts with HF and compared with 20 healthy people	Poincaré plot is a semi-quantitative tool which can be applied to the analysis of R-R interval
Laitio et al., 2002 [22]	HRV and Poincaré plots of 40 pts with CABG	SD1/SD2 ratio is the most powerful independent predictor of postoperative ischaemia
Laitio et al., 2004 [13]	32 pts aged ≥60 yrs admitted to hospital for surgical repair of traumatic hip fracture	Alpha-1 predicts post operative myocardial infarction
Lombardi et al.1996 [8]	HRV in 2 groups of pts after MI (normal and reduced LVEF). Group1: 20 pts; Group 2: 15 pts	Steeper slope of the negative regression line between power and frequency among reduced LVEF
Mäkikallio et al., 1998 [23]	38 pts with stable angina without previous MI or cardiac medication and 38 age matched healthy pts	Alpha-1 helps differentiate CAD and healthy pts

## Glossary of Abbreviations

Alpha-1 (α1): short-term fractal scaling exponent, a non-linear measure of the randomness or correlatedness of heart rate patterns
CABG: coronary artery bypass grafting
DFA1: same as alpha-1
HRV: heart rate variability
SDNN: standard deviation of all normal-to-normal interbeat intervals in ms, a traditional measure of heart rate variability
SD1: short axis of ellipse fitted around a Poincaré plot of N-N intervals in ms
SD2: long axis of an ellipse fitted around a Poincaré plot of N-N intervals in ms
SD12: SD1/SD2
